# Health Tract, No. 286

**Published:** 1867-04

**Authors:** 


### BREAD MAKING.

Bread is the staff of life literally. The French make the best in the world, one secret being that the dough is put into the oven at a high temperature, at which it is kept, marked by a thermometer, until it is done. It is however known, that other things being equal, the more thoroughly the dough is kneaded, the better will be the bread. Bad Bread puts nameless thousands of gold into the doctors pockets every year, and hurries multitudes into an untimely grave. The following method, from that excellent and popular paper, at Albany, New York, *The Country Gentleman*, is one of the very best.

"Take six good-sized potatoes, wash and pare them, and boil in two quarts of water, aud with them a handful of hops, in a small bag kept for the purpose. When quite soft take them out, mash fine, and pour upon them the water in which they were boiled, adding a little water to make up for what may have boiled away, and also half a cup of salt and same of white sugar. When cooled down to a lukewarm temperature, add one cup of yeast to ferment it with. I do not say raise, for it does not rise; it works like beer, and having been covered closely and kept in a warm place, in the course of five or six hours the entire surface will be covered with fine bubbles, which indicates that it is ready for use. It should now be bottled and put in the cellar, where it will keep a long time. The bottles must not be corked tight at first, or they will be liable to burst. If the theory be true that some of the same kind must be used to start with, some difficulty may be encountered in introducing it where it is not used. I was furnished with some of the right sort in the beginning, and therefore had no occasion to experiment; and, though I have been gravely assured by those who have done so that no other would answer, still I would do so if occasion required. One cup of yeast is sufficient for three common-sized loaves, the requisite quantity of flour for which every cook knows. I usually mix my bread with milk, because I like the taste better, although I have made lighter, nicer looking bread with water than with milk.

"Very good cooks say they would prefer water to milk for the purpose even at the same cost; that is a matter merely of taste. I prefer to mix it up at night in order to bake it in the early part of the day, stirring it until it is as stiff as it can be stirred with a spoon, liking this method better than setting a soft sponge, as some cooks do. In the morning, the temperature having been favorable, my pan is filled. I then take it out upon a board, and knead it thoroughly, alternately chopping it with a chopping-knife, and working on more flour as long, I had almost said, as I can get any in. If made with water, I work in a little shortening; if with new milk, none is necessary. In this thorough kneading process lies one secret in bread making, the advantages of which some cooks do not know, or knowing, do not care to profit by. Some prefer to dip their dough into the tins with a spoon. Well, let them do so, but the sequel does not show such bread as is needed (kneaded,) spell the word as you will.

"Having accomplished this part of the process, I return it to the pan and let it rise the second time. I then take it out, form it into smooth loaves, let them rise until the tins are rounding full, and bake. 'For the length of time required for baking,' I have no rule; that of some is just one hour. I bake my bread until it is 'done,' let the time required be shorter or longer."

